# Efficacy of air polishing in comparison with hand instruments and/or power-driven instruments in supportive periodontal therapy and implant maintenance: a systematic review and meta-analysis

**DOI:** 10.1186/s12903-022-02120-6

**Published:** 2022-03-23

**Authors:** Shiuan Lee Tan, Galvinderjeet Kaur Grewal, Nor Shafina Mohamed Nazari, Tuti Ningseh Mohd-Dom, Nor Adinar Baharuddin

**Affiliations:** 1 Department of Restorative Dentistry, Faculty of Dentistry, Universiti Malaya, Lembah Pantai, 50603 Kuala Lumpur, Malaysia; 2 Department of Family Oral Health Faculty of Dentistry, Universiti Kebangsaan Malaysia, Jalan Raja Muda Abdul Aziz, 50300 Kuala Lumpur, Malaysia

**Keywords:** Systematic review, Air polishing, Supportive periodontal therapy, Implant maintenance

## Abstract

**Background:**

Supportive periodontal therapy (SPT) is the key for a stable periodontal health following active treatment. Likewise, implant maintenance is crucial following implant placement. This systematic review aimed to assess clinical outcomes, patients’ perception, and cost-effectiveness of repeated periodontal therapy with air polishing devices (APDs) in comparison with hand instruments and/or power-driven instruments (conventional interventions) in SPT and implant maintenance.

**Methods:**

Electronic search for randomised controlled clinical trials with minimum 6 months follow-up for SPT and implant maintenance programme was conducted for data published from 01 January 2000 to 30 April 2020 using multiple databases and hand searching. Risk of bias was assessed using the Revised Cochrane Risk-of-Bias tool (RoB 2).

**Results:**

A total of 823 articles were screened. 4 SPT and 2 implant maintenance studies were eligible for inclusion. For SPT, repeated APDs interventions revealed no statistically significant difference when compared to the conventional interventions (weighted mean difference [WMD] 0.11 mm, p = 0.08). Likewise, no statistical difference was noted in terms of percentage of bleeding on probing (BOP) and clinical attachment level (CAL) gain. APDs were associated with lower pain score (based on Visual Analogue Scale) and higher patient acceptance in SPT studies. For implant maintenance, APDs resulted in reduction in PPD and percentage of BOP. However, CAL gain was comparable between the two groups. In terms of patient reported outcomes, no implant maintenance studies recorded any forms of patient reported outcomes. In addition, no studies reported on economic evaluation of APDs in both SPT and implant maintenance.

**Conclusion:**

Within the limitations of this systematic review and meta-analysis, repeated subgingival debridement using APDs in SPT resulted in similar clinical outcomes but better patients’ comfort when compared to the conventional interventions. For implant maintenance, there is limited evidence to show that repeated application of APDs leads to improved clinical outcomes when compared to conventional treatments.

**Supplementary Information:**

The online version contains supplementary material available at 10.1186/s12903-022-02120-6.

## Background

Maintenance phase is necessary following active therapy. With regards to maintenance phase following active periodontal therapy, it has been recommended for periodontal patients to adhere to supportive periodontal care. The recall interval suggested by the S3 level clinical practice guidelines for treatment of Stage I–III periodontitis was 3 to 12 months, depending on individual’s risk profile and periodontal conditions [1]. In terms of implant maintenance interval, it must also be tailored to patient’s risk profile and a minimum recall interval of 5 to 6 months had been recommended [2]. Nevertheless, a systematic review involving studies with diverse interval periods, the longest being a 12-month duration, was unable to establish a definite timepoint for recall interval [3]. Hence, the authors suggested a periodic implant maintenance at least annually can potentially improve peri-implant health in relation to survival rate, peri-implant mucositis and peri-implantitis [3]. Absence of implant maintenance may increase risks for peri-implant diseases [4, 5].

During maintenance phase, conventional modality such as debridement by hand and/or ultrasonic instruments is used to remove biofilm [6]. However, repeated debridement may cause irreversible microscopic damage to the tooth surfaces [7, 8]. Inadvertent removal of these surfaces may encourage biofilm deposition and in case of root surfaces, sensitivity can be resulted [9].

Given that repeated debridement is anticipated during the maintenance phase, a more surface friendly modality such as air polishing devices (APDs) may be appropriate. APDs has been said to be more comfortable than the conventional debridement modality [10]. The effectiveness of APDs versus conventional hand instruments and/or power-driven scalers, both on natural dentition or on dental implants has been studied widely [10–21].

There was also a number of previous systematic reviews investigating the effects of APDs on oral tissues and patient perception towards the devices in SPT subjects. However, the reviews reported on studies related to debridement of a single application as well as on earlier and less-refined air polishing powders [22–25]. In addition, the efficacy of APDs on clinical outcomes and patients’ perception in SPT subjects was limited to short evaluation period [24]. No reviews had reported on the efficacy of APDs in implant maintenance subjects. Thus, there is a clear uncertainty on repeated use of APDs in SPT and implant maintenance patients.

Other than clinical outcomes and patients’ perception, the economic evaluation associated with APDs should be investigated since the acquired price for these devices is more expensive than the conventional devices. Should APDs be proven as a better alternative to the conventional modality, the cost of maintenance phase may have a negative economic impact on the patients and public healthcare funding.

Therefore, the aim of this review was to critically and comprehensively evaluate the effect of repeated use of APDs in comparison with hand instruments and/or power-driven instruments, as well as the cost-effectiveness of these devices in SPT and implant maintenance.

## Methods

### Focused questions

1. Does repeated intervention using APDs provide superior clinical, and patient reported outcomes as well as cost-effectiveness compared to conventional debridement using hand scaling and/or power-driven instruments in SPT patients?
2. Does repeated intervention using APDs provide superior clinical, and patient reported outcomes as well as cost-effectiveness compared to conventional debridement using hand scaling and/or power-driven instruments in implant maintenance patients?

### Objectives

- To determine and compare clinical outcomes of debridement using APDs compared to hand scaling and/or power-driven instruments in SPT and implant maintenance patients,
- To determine and compare patients’ perception during treatment with APDs in comparison to hand scaling and/or power-driven instruments in SPT and implant maintenance patients,
- To determine and compare the cost-effectiveness of APDs compared to hand scaling and/or power-driven instruments in SPT and implant maintenance patients.

Prior to commencing the review, the protocol was registered on the PROSPERO database (www.crd.york.ac.uk/PROSPERO) with registration number CRD42020190664. Besides, this review had been prepared following the PRISMA statement (Additional file 1) for reporting systematic review [26] and Cochrane Handbook of Systematic Reviews of Interventions [27]. The PICO (Population, Intervention, Comparison, Outcomes) framework was used to develop the focused questions.

#### PICO for focused question 1

- Population: SPT patients, aged ≥ 18 years, with good systemic health or controlled systemic diseases
- Intervention: Use of APDs for non-surgical supra- and/or subgingival therapy
- Comparison: Conventional hand or sonic/ultrasonic instruments or a combination of both
- Outcomes: Primary outcomes were assessment on changes of clinical parameters such as Probing Pocket Depth (PPD), Clinical Attachment Level (CAL) and Bleeding on Probing (BOP). Secondary outcomes were Patient Reported Outcome Measures (PROMs) in terms of Visual Analogue Scale (VAS) score, questionnaires on Quality of Life (QoL) or patient interview, as well as economic evaluation using cost effectiveness analysis, cost utility analysis or cost benefit analysis.

#### PICO for focused question 2

- Population: Implant maintenance patients, aged ≥ 18 years, with good systemic health or controlled systemic diseases
- Intervention: Use of APDs for non-surgical supra- and/or subgingival therapy
- Comparison: Conventional hand or sonic/ultrasonic instruments or a combination of both
- Outcomes: Primary outcomes were assessment on changes of clinical parameters such as PPD, CAL and BOP. Secondary outcomes were PROMs in terms of VAS score, questionnaires on QoL or patient interview, as well as economic evaluation using cost effectiveness analysis, cost utility analysis or cost benefit analysis.

### Types of studies

Only randomised controlled clinical trials (RCTs) with minimum 6 months follow-up for SPT and implant maintenance programme were eligible for inclusion in this review. The full-text articles were evaluated to determine if the articles met the inclusion criteria specified below.

### Inclusion criteria

- RCTs in SPT phase (focused question 1) or implant maintenance programme (focused question 2).
- Adult subjects of ≥ 18 years of age.
- Individuals in good systemic health or controlled systemic diseases.
- Intervention / test group using APDs; control with hands instrumentation and/or sonic/ultrasonic scalers.

### Exclusion criteria

- Lack of repeated interventions or retreatment in periodic recall visits.
- Pregnant and lactating females.
- Antibiotic usage within the last four weeks before the trial.

### Search strategy

A highly sensitive search of electronic databases including Cochrane library, MEDLINE, Web of Science, EMBASE, as well as Dentistry and Oral Sciences Source, was conducted to identify relevant articles published in English language from 01 January 2000 to 30 April 2020 using a string of medical subject headings and free-text terms. OpenGrey was searched for unpublished, grey literature. The electronic search was complemented by a hand search of publications relating to the review topic from the Journal of Clinical Periodontology, Journal of Periodontology, Clinical Implant Dentistry and Related Research, as well as Clinical Oral Implant Research. Furthermore, the cited references from included full-text articles and related systematic reviews were screened. The search strategy was adapted and revised accordingly for each online database mentioned above (Additional file 2). The last date of search was 5 May 2020.

### Study selection

First, duplication of studies due to repeated citations in different databases were removed. Following this, titles and abstract of the studies identified in the searches were screened by two review authors (TSL and GKG), in duplicate and independently. Subsequently, the full text of all the publications that met the inclusion criteria or for which there was insufficient information were obtained. Unrelated publications were excluded at this point. Full text of potentially relevant articles was then downloaded and assessed for eligibility based on the inclusion and exclusion criteria. A third reviewer (NAB) adjudicated the disagreement that occurred, and the final selection was mutually agreed upon by all three assessors.

### Unclear or missing data

Exclusion of any further studies was recorded with explanations for rejection. Efforts were made to contact the corresponding authors through e-mails, should there be any incomplete data or missing information for further clarification as well as to acquire full-text articles if only abstracts were found.

### Data extraction and management

Study details were collected using a form specifically designed for data extraction for this review. Two reviewers independently extracted the following information:

- First author’s name and year of publication, study location (country).
- Study population including setting, number of patients, mean age, gender, and smoking status.
- Study design, duration of follow-up and case definition of study sites, if any.
- Details on the treatment groups/interventions, including but not limited to:
  - Type of powder used, with or without special nozzle.
  - Type of conventional instrument.
  - Time allocation on each site.
  - Retreatment interval.
- Details of included variables such as clinical parameters, patient reported outcome measures (PROMs), economic evaluation as well as timepoints of assessment.
- Details of corresponding treatment outcomes.

Moreover, based on available outcomes reported in each study, continuous data including clinical parameters (PPD, CAL and BOP) and PROMs (VAS) were extracted in the form of mean and standard deviation (SD) and tabulated according to treatment groups, baseline, and follow-up comparisons.

### Quality assessment

Risk of bias was assessed using the Revised Cochrane Risk-of-Bias tool (RoB 2) [28]. The tool evaluates five domains of bias, comprising of (i) randomisation, (ii) deviations from intended interventions, (iii) missing data, (iv) outcome measurements and (v) selective reporting. Overall risk-of-bias judgement of each included study could be classified as low, some concerns or high, based on prespecified criteria. Inter-examiner reliability was assessed using Cohen’s Kappa statistics. Disagreements were resolved and consensus was reached by discussion or consulting a third reviewer (NAB).

### Data synthesis

All SPT and implant maintenance studies were qualitatively evaluated through narrative synthesis. Meta-analysis was performed and the outcomes were mean difference as well as standard deviation (SD). For studies where standard error of the mean (SEM) were reported instead, SDs were obtained by multiplying the SEM with the square root of the sample size (SEM = SD/√N) [29]. When the SEMs or SDs in the studies were reported only for baseline and follow up mean values but not for the mean difference from baseline, the SD for the mean difference was imputed from the existing data by presuming a correlation coefficient of 0.8 between the baseline and final mean values [29].

For studies with similar follow up period, a random-effect meta-analysis model by DerSimonian and Laird with inverse-variance approach was used in view of expected heterogeneity between studies [30]. The meta-analysis was performed using RevMan Version 5.4, aimed to integrate the findings of similar studies in terms of clinical parameters including PPD and CAL. The effect size was expressed as weighted mean difference (WMD) and SDs of the WMD with 95% confidence intervals. Heterogeneity across studies was measured using I^2^ statistic test.

However, meta-analyses for BOP and PROMs (VAS) in SPT as well as the clinical outcomes and VAS from implant maintenance studies were not feasible due to inadequate data, different study methodologies and finite number of clinical trials comparing APDs with conventional therapy.

## Results

### Search

**Fig. 1:**
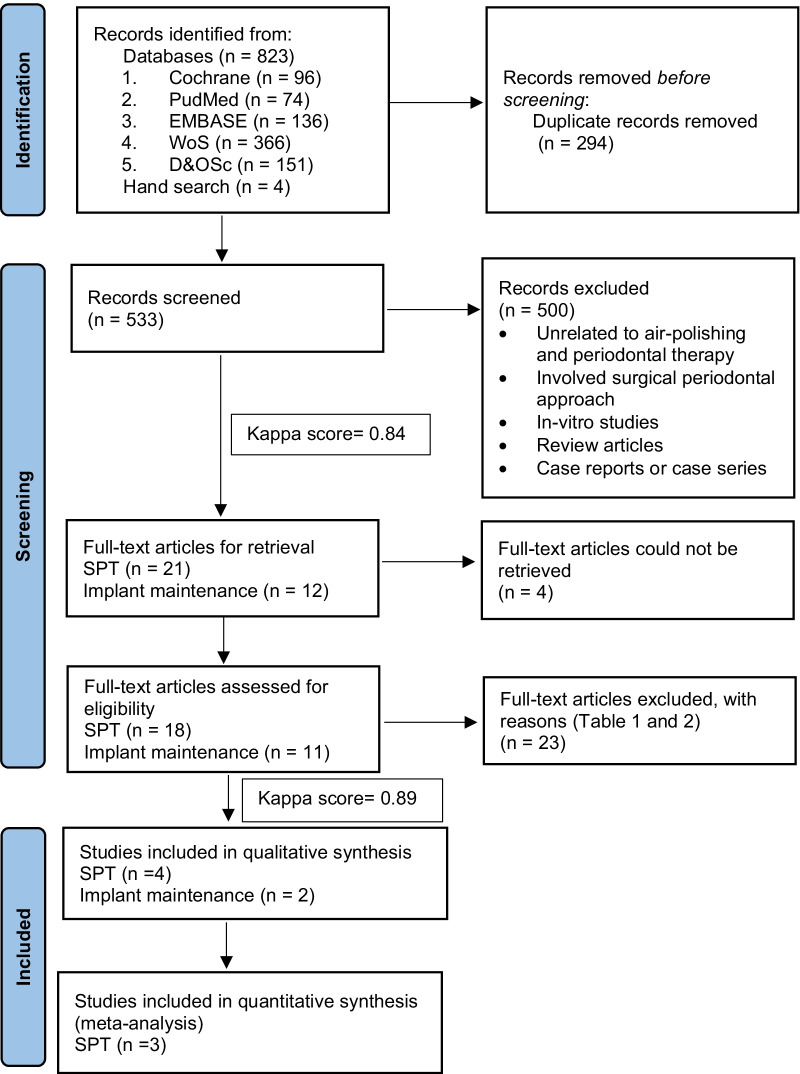
PRISMA 2020 flow diagram summarising the study selection process

Searching of the databases and trial registry yielded a total of 827 records. Following removal of duplicates, the titles and abstracts of 533 records were screened. Finally, 33 records that were identified for full-text articles were retrieved. Only 29 full-text articles were assessed for eligibility based on inclusion and exclusion criteria. Another 23 full-text articles were excluded for reasons such as non-English publication, lack of clinical data, absence of repeated therapy, studies other than human controlled clinical trials and treatment of peri-implant diseases (Tables 1 and 2). Figure 1 describes the screening process. In total, 6 studies; 4 SPT and 2 implant maintenance were accepted for the review. Inter-reviewer agreement for retrieval and eligibility assessment were excellent (kappa scores = 0.84 and 0.89, respectively).

**Table 1:** Reasons for exclusion (SPT studies)

Reasons for exclusion	SPT studies
In Chinese	Hu et al. [31]
In Chinese	Zhao et al. [32]
In German	Moene et al. [33]
Same cohort as one of the included studies—pilot study	Kruse et al. [34]
Lack of information on clinical data	Petersilka et al. [35]
Lack of information on clinical data	Petersilka et al. [36]
Lack of repeated interventions / retreatment	Flemmig et al. [12]
Lack of repeated interventions / retreatment	Hagi et al. [37]
Lack of repeated interventions / retreatment	Lu et al. [16]
Lack of repeated interventions / retreatment	Lu et al. [38]
Lack of repeated interventions / retreatment	Moene et al. [10]
Lack of repeated interventions / retreatment	Simon et al. [39]
Lack of repeated interventions / retreatment	Wennstrom et al. [20]
Control group using water irrigation only	Sekino et al. [40]

**Table 2:** Reasons for exclusion (implant maintenance studies)

Reasons for exclusion	Maintenance studies
Book chapter	Monje et al. [41]
In German	Petersilka et al. [42]
Observational study with no comparator	Duarte et al. [43]
Observational study with no comparator	Heitz-Mayfield et al. [44]
Treatment of peri-implant diseases	Al Ghazal et al. [11]
Treatment of peri-implant diseases	Schmidt et al. [19]
In vitro study	Koishi et al. [45]
Lack of repeated interventions / retreatment	Menini et al. [46]
Lack of repeated interventions / retreatment	Mussano et al. [47]

### Study characteristics

All studies recruited adult subjects and were carried out in a single centre university setting except for one multicentre study [21] which involved seven dental practices with at least eight subjects from each practice. Mean age for the study population was between 52.5 years to 59.7 years. Further study characteristic is summarised in Tables 3 and 4.

**Table 3:** Key characteristics of SPT studies

Study	Population	Study design	Treatment groups	Variables	Treatment outcome
Hagi et al. [13]	Setting: University	RCT	**Test**: EPAP with a single-use nozzle (5 s each site)	**Primary**: Site-specific BOP	Mean BOP:
		Parallel			Test = 40.45%; Control = 42.53%
	40 SPT subjects	Examiner-masked		**Clinical**: Full mouth and site-specific PI, BOP, PPD and CAL	
Switzerland	(38 completed study)		**Control**: Hand instruments only, no time limit		Mean PPD reduction:
		Duration: 6 months			Test = 0.67 mm; Control = 0.68 mm
	Mean age: 54.5 y			**Safety assessment**: Adverse events at every visit by clinical examination and patient interview	
	Gender: 15 F, 25 M	Study sites: BOP and PPD of ≥ 4 mm without presence of detectable subgingival calculus, exclude furcation involved and adjacent test sites	Without local anaesthesia		EPAP and curettes resulted in significant but similar reductions of clinical parameters. No statistical difference between both groups for site specific BOP, PPD and CAL
			**Retreatment:** 3-month		
	Smoking status: Included but not detailed			**Timepoints**: Baseline, 6-month	No adverse events reported
Kargas et al. [14]	Setting: University	RCT	**Negative control**:	**Primary**: PPD change	Mean PPD reduction:
		Split mouth	1) Subgingival GPAP—5 s per site		GPAP = 0.26 mm; UD = 0.66 mm; SRP = 0.44 mm
	25 SPT subjects	Blinding not mentioned		**Clinical**: PPD, CAL, GR, GI, PI	
Greece			2) Subgingival ultrasonic debridement (UD)		
	Mean age: 52.5 y	Duration: 6 months		**PROMs**: Pain perception, cold or pressure (questionnaire at baseline after treatment)	GPAP group had significantly higher PPD than the SRP group at 1,3 and 6 months and higher level of CAL at 1 month. No differences among groups for GR, GI and PI
			3) No further subgingival treatment		
	Gender: 10 F, 15 M	Study site: No BOP and PPD > 4 mm, furcation not specified as an exclusion criterion			
			**Positive control**: Subgingival scaling with hand instruments (SRP)		
	Smoking status: Non-smoker			**Timepoints**: baseline, 1-, 3- and 6-month	
					Less pain, no sense of pressure with GPAP
			**Retreatment**: 3-month		
Kruse et al. [15]	Setting: University	RCT	**Test**: APD with trehalose powder and single-use nozzle (total 20 s)	**Primary**: PPD change	Mean PPD reduction:
		Split mouth			Test = 1.86 mm; Control = 1.87 mm
	44 SPT subjects	Examiner-masked		**Clinical**: PPD, CAL, GR, BOP, PI, SBI	
Germany					APD and sonic device resulted in significant intra-group reduction of PPD, CAL and BOP after 6 months with no significant inter-group differences
	Mean age: 59.7 y	Duration: 6 months	**Control**: Sonic scaler (total 20 s)		
				**PROMs**: VAS score after treatment for each procedure	
	Gender: 18 F, 26 M	Study site: Single-rooted teeth with PPD 5 mm and BOP or PPD > 5 mm ± BOP			
			**Retreatment:** 3-month		
	Smoking status: Included but not detailed			**Timepoints**: Baseline, 3- and 6-month	A significant lower incidence of discomfort for air polishing compared to sonic scaling
Muller et al. [18]	Setting: University	RCT	**Test**: EPAP with a single-use nozzle (5 s each site)	**Primary**: Presence or absence of PPD > 4 mm per subject	Mean *n* sites with PD > 4 mm:
		Split mouth			Test = 3.6; Control = 3.9
	50 SPT subjects	Examiner-masked			
Switzerland	(49 completed study)		**Control**: Ultrasonic scaler (20 s per site)	**Clinical**: PPD, GR, BOP, PI, root hypersensitivity	The number of pockets > 4 mm per subject, PPD and BOP were significantly lower at month 12 with no significant difference between EPAP therapy and ultrasonic debridement
		Duration: 12 months			
	Mean age: 58.5 y				
		Study site: PPD > 4 mm with absence of clinically detectable subgingival calculus, furcation not specified as an exclusion criterion	**Retreatment:** 3-, 6- and 9-month	**PROMs**: VAS score after each procedure	
	Gender: 29 F, 21 M				
	Smoking status: Included but not detailed			**Timepoints**: Baseline and 12-month	A significant difference in favour of air-polishing for pain / discomfort

*APD* air-polishing device; *BOP* bleeding on probing; *CAL* clinical attachment level; *EPAP* erythritol powder air-polishing; *F* female; *GI* gingival index; *GPAP* glycine powder air-polishing; *GR* gingival recession; *M* male; *PI* plaque index; *PPD* probing pocket depth; *PROMs* patient reported outcome measures; *RCT* randomised controlled clinical trial; *SBI* sulcular bleeding index; *SPT* supportive periodontal therapy; *VAS* visual analogue scale

**Table 4:** Key characteristics of implant maintenance studies

Study	Population	Study design	Case definition	Treatment groups	Variables	Treatment outcome
Lupi et al. [17]	Setting: University	RCT	No signs of inflammation or mucositis	**Test**: GPAP with Perio-Flow nozzle (5 s each site)	**Primary**: PPD change	Mean PPD reduction: Test = 0.64 mm; Control = − 0.31 mm
		Parallel				
	46 subjects (35 partial; 11 total edentulism) with 88 implants	Examiner-masked	No PPD ≥ 4 mm and suppuration; No bone resorption ≥ 30% compared to initial situation; No implant mobility		**Clinical**: PI, BOP, PPD, CAL, bleeding score	
Italy				**Control**: Plastic curettes + irrigation with 0.1% CHX + submucosal application 1% CHX gel		GPAP statistically improved PPD, PI, BOP and bleeding score after 6 months; more effective than Control in maintaining the peri-implant health of PPD. No significant changes of CAL in both groups
		Duration: 6 months				
	Mean age: 54.2 y				**PROMs**: Not reported	
	Gender: Not reported			Without local anaesthesia		
					**Timepoints**: Baseline, 3- and 6-month	
	Smoking status: Non-smokers		At least 2 mm keratinized peri-implant mucosa	**Retreatment:** at monthly basis		
Ziebolz et al. [21]	Setting: Multicentre study (7 dental practices)	RCT	Not specified	**Test (Adjunctive AP)**:	**Primary**: Not reported	No significant implant-related differences in PPD, MR and BOP in group 2), 3) and 4) between baseline and follow-up, while group 2) showed a significant difference in PPD
		Parallel		1) Curette + GPAP + prophylaxis brush		
		Masking not mentioned	Study began after prosthetic restoration of a previously inserted implant with no signs of inflammation, no previous non-surgical or surgical therapy		**Clinical**: Papillary bleeding index, approximal PI, PPD, MR, BOP	
Germany	62 subjects (partially or fully edentulous) with 101 implants			2) Curette + GPAP + prophylaxis brush + CHX varnish		
		Duration: 12 months				
	Mean age: 55.21 ± 11.3 y			**Control (Adjunctive Sc)**:	**PROMs**: Not reported	
				3) Curette + sonic scaler + prophylaxis brush		
					**Timepoints**: baseline and 12-month	
	Gender: 27 F, 35 M			4) Curette + sonic scaler + polishing with prophylaxis brush + CHX varnish		
	Smoking status: Non-smoker					
				**Retreatment**: 3-, 6-, 9-month		

*AP* air-polishing; *BOP* bleeding on probing; *CAL* clinical attachment level; *CHX* chlorhexidine; *F* female; *GI* gingival index; *GPAP* glycine powder air-polishing; *M* male; *MR* mucosal recession; *PI* plaque index; *PPD* probing pocket depth; *PROMs* patient reported outcome measures; *RCT* randomised controlled clinical trial; *Sc* scaling; *SPT* supportive periodontal therapy; *VAS* visual analogue scale

### Sample characteristics

These studies encompassed 156 SPT and 108 implant maintenance patients who had successfully completed the clinical trials within the specified timeframes in each study. Among the implant study population, the subjects were either partially or fully edentulous with a total of 189 dental implants evaluated. The key characteristics of the included studies are detailed in Tables 3 and 4.

### Risk of bias and methodologic quality

**Fig. 2:**
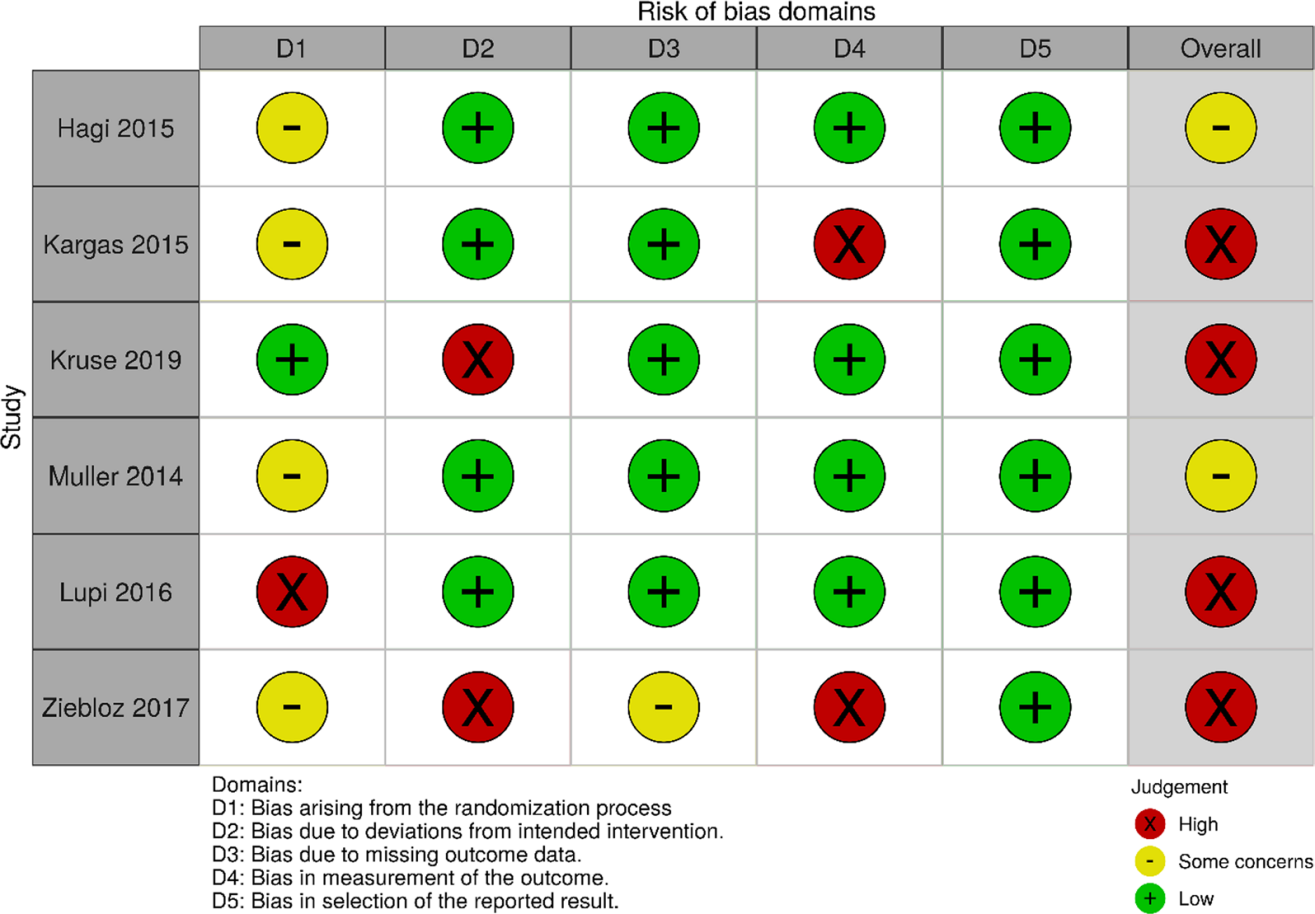
Risk of bias summary of each included study

The Revised Cochrane RoB-2 tool (Fig. 2) was selected to evaluate the risk of bias and to determine the internal validity of the selected studies. Studies by Hagi et al. [13] and Muller et al. [18] were considered to have some concerns of bias. While studies by Kargas et al. [14]; Kruse et al. [15]; Lupi et al. [17]; and Ziebolz et al. [21] were regarded as high risk of bias.

### Primary outcomes comparison: clinical parameters

1. Probing Pocket Depth (PPD)

PPD was evaluated in all SPT and implant maintenance studies. Generally, SPT studies reported statistically significant PPD reduction after repeated debridement with APDs after 6 months [13, 15] or 12 months [18]. However, there was no statistically significant difference between test (APDs) and control (hand instruments and/or power-driven instruments) groups except in one study that favoured the control treatment [14].

For implant maintenance, Lupi et al. [17] reported that the use of APDs significantly reduced PPD after 6 months. Moreover, the mean PPD values were statistically significantly different between the test and control groups both at 3- and 6-month. On the other hand, Ziebolz et al. [21] revealed significantly higher PPD was observed at follow-up in the group receiving adjunctive APDs, in particular using glycine powder air polishing (GPAP). Nevertheless, observation at implant-level revealed no statistically significant inter-group differences over the study period.

2. Clinical Attachment Level (CAL)

4 out of 6 studies recorded CAL. In SPT studies, Hagi et al. [13] and Kruse et al. [15] found statistically significant gain in CAL for both test (APDs) and control treatment groups between baseline and at 6 months visits, with no significant inter-group differences. On the other hand, Kargas et al. [14] indicated that there was no significant CAL gain in the APD group after 6 months in comparison to baseline but statistically significant inter-group differences in all time points.

For implant maintenance, Lupi et al. [17] observed a non-significant CAL gain at 3 and 6 months compared to baseline in the test group. There were no significant CAL changes in both test and control groups, as well as inter-group differences after 6 months.

3. Bleeding on Probing (BOP)

In general, three SPT studies revealed statistical significant intra-group reduction of BOP percentage in test and control groups at 6 months [13, 15] and 12 months [18] with no significant inter-group differences except for one study [15].

For implant maintenance, Lupi et al. [17] reported a statistically significant decrease in percentage of BOP in test and control groups at 6 months, along with significant inter-group differences. Conversely, Ziebolz et al. [21] documented no statistically significant intra- and intergroup differences from baseline to 12 months for all treatment groups.

### Secondary outcomes comparison

1. Patient Reported Outcome Measures (PROMs)

Only 3 SPT studies reported on patients’ perception during treatment. Pain perception was evaluated using the VAS (0 to 10 scale) following each procedure in two SPT studies [15, 18]. The results showed treatment with APDs led to significant lower pain compared to power-driven instruments. Kargas et al. [14] assessed patients’ perception using a questionnaire in which information on pain perception (0 to 4 scale), cold and pressure during treatment as well as individual’s preferred technique of treatment were documented. Subjects mainly reported less pain, no sense of pressure and perceived treatment with APD being more friendly. Unfortunately, none of the implant maintenance studies recorded any form of patient reported outcome.

2. Economic evaluation

Regrettably, none of the included studies assessed and reported on this outcome.

Data comparisons for each study are summarised in Tables 5 and 6.

**Table 5:** Comparison of clinical parameters and VAS between intervention and comparators in SPT studies

Parameter	Study	Intervention /	Total	Baseline /	*P*	Follow up / mean (SD)					
		comparator(s)		mean (SD)		3-month	*P*	6-month	*P*	12-month	*P*
Mean PPD	Hagi et al. [13]	APD (EPAP)	91	4.46 (0.67)	> 0.05			3.78 (1.23)	> 0.05		
(mm)		Curettes	96	4.65 (0.88)				3.92 (1.40)			
	Kargas et al. [14]	APD (GPAP)	25	4.78 (0.50)	NS	4.40 (0.55)		4.52 (0.45)			
		Ultrasonics	25	4.66 (0.50)		3.84 (0.35)	*	4.00 (0.40)	*		
		Curettes	25	4.50 (0.45)		3.70 (0.40)	*	4.06 (0.50)	*		
	Kruse et al. [15]	APD (TPAP)	44	5.52 (0.93)	NS	4.25 (1.12)	> 0.05	3.66 (0.81)	> 0.05		
		Sonic	44	5.55 (0.90)		4.11 (1.08)		3.68 (0.86)			
	Muller et al. [18]	APD (EPAP)	50	5.2 (0.4)	0.003					4.5 (1.0)	NS
		Ultrasonics	50	5.4 (0.6)						4.4 (1.1)	
Mean CAL	Hagi et al. [13]	APD (EPAP)	91	4.90 (1.81)	> 0.05			4.43 (2.26)	> 0.05		
(mm)		Curettes	96	5.07 (2.06)				4.37 (2.43)			
	Kargas et al. [14]	APD (GPAP)	25	5.42 (0.65)		5.38 (0.60)		5.40 (0.55)			
		Ultrasonics	25	5.12 (0.55)	NS	4.76 (0.55)	*	4.82 (0.55)	*		
		Curettes	25	4.94 (0.45)	*	4.84 (0.45)	*	4.82 (0.45)	*		
	Kruse et al. [15]	APD (TPAP)	44	6.93 (1.50)	NS	5.80 (1.65)	> 0.05	5.30 (1.52)	> 0.05		
		Sonic	44	7.27 (1.80)		6.00 (1.73)		5.84 (1.71)			
BOP (%)	Hagi et al. [13]	APD (EPAP)	FM	31.70 (14.24)	> 0.05			26.11 (17.88)	> 0.05		
				36.45 (17.51)				27.89 (15.53)			
	Kruse et al. [15]	APD (TPAP)	T	86.36	NS	59.09	< 0.001	40.91	< 0.001		
		Sonic		88.64		63.64		34.09			
	Muller et al. [18]	APD (EPAP)	S	58 (50)	NS					31 (47)	NS
		Ultrasonics		48 (50)						27 (45)	
VAS (1–10)	Kruse et al. [15]	APD (TPAP)		2.33 (2.14)	< 0.001						
		Sonic		4.91 (2.65)							
	Muller et al. [18]	APD (EPAP)		2.04 (2.17)	0.004						
		Ultrasonics		4.86 (2.92)							

^*^Statistical significance between APD and comparator and other groups (Bonferroni's test)

*NS* not significant; *FM* full-mouth; *T* tooth; *S* site; *APD* air polishing device; *EPAP* erythritol powder air polishing; *GPAP* glycine powder air polishing; *TPAP* trehalose powder air polishing; *PPD* probing pocket depth; *CAL* clinical attachment level; *BOP* bleeding on probing; *VAS* visual analogue scale

**Table 6:** Comparison of clinical parameters and VAS between intervention and comparators in implant maintenance studies

Parameter	Study	Intervention/comparator(s)	Total	Baseline/mean (SD)	*P*	Follow up/mean (SD)					
						3-month	*P*	6-month	*P*	12-month	*P*
Mean PPD	Lupi et al. [17]	GPAP	24	2.51 (0.24)	NS	2.19 (0.35)	< 0.05	1.87 (0.38)	< 0.001		
(mm)		MDA	22	2.39 (0.46)		2.54 (0.48)		2.70 (0.37)			
	Ziebolz et al. [21]	Adjunctive GPAP	45	1.77 (1.58)	NR					2.31 (1.54)	NR
		Adjunctive GPAP + CHX	37	2.00 (1.38)						2.05 (1.32)	
		Adjunctive Sc	36	1.75 (1.23)						2.21 (1.32)	
		Adjunctive Sc + CHX	49	2.67 (1.63)						2.23 (1.28)	
Mean CAL	Lupi et al. [17]	GPAP	24	1.06 (1.07)	NS	1.03 (1.09)	NS	0.89 (1.04)	NS		
(mm)		MDA	22	0.55 (0.87)		0.63 (0.94)		0.74 (0.96)			
BOP (%)	Lupi et al. [17]	GPAP	I	45.83 (39.47)	< 0.001	33.33 (32.69)	0.05	20.83 (30.99)	< 0.01		
		MDA		84.09 (25.05)		71.59 (27.05)		70.45 (26.32)			
	Ziebolz et al. [21]	Adjunctive GPAP	I	11.5	NR					11.5	NR
		Adjunctive GPAP + CHX		4.8						1	
		Adjunctive Sc		0						4.2	
		Adjunctive Sc + CHX		0						100.25	

NS not significant; *NR* not reported; *I* implant-level; *GPAP* glycine powder air polishing; *Sc* Sonic Scaling; *CHX* chlorhexidine varnish; *MDA* manual debridement and chlorhexidine administration treatment group; *PPD* probing pocket depth; *CAL* clinical attachment level; *MR* mucosal recession; *BOP* bleeding on probing; *BS* bleeding score

### Meta-analysis of primary outcomes

**Fig. 3:**
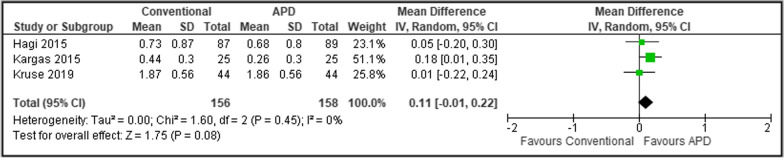
Forest plot for mean PPD reduction in SPT

**Fig. 4:**
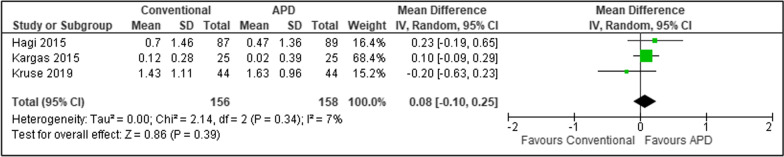
Forest plot for mean CAL gain in SPT

Meta-analyses (Figs. 3 and 4) were performed on SPT studies with similar follow up period of 6 months. Muller et al. [18] was excluded due to different follow up period. The results showed that repeated treatment using APDs had statistically non-significant PPD reduction than repeated treatment with conventional means throughout the study duration of 6 months (WMD 0.11, 95% confidence interval [CI] − 0.01 to 0.22, p = 0.08, I^2^ = 0%). In addition, there was no statistically significant difference in CAL gain between APD and conventional treatment (WMD 0.08, 95% CI − 0.10 to 0.25, p = 0.39, I^2^ = 7%). A very low level of heterogeneity was noted in both analyses.

## Discussion

This systematic review was conducted to determine the effects of repeated periodontal therapy with APDs in comparison to hand instruments and/or power-driven instruments in a population of patients receiving SPT and implant maintenance care with at least six months follow-up. The primary outcome was the change in clinical parameters such as PPD, CAL and BOP at various time points. The secondary outcomes were on PROMs and economic effectiveness.

### Key findings in SPT studies

#### Clinical parameters

In general, APDs and conventional therapy resulted in statistically significant reduction in PPD and BOP percentage as well as gain in CAL. Most studies reported no statistically significant differences in clinical outcomes between both treatment modalities after repeated interventions. Due to inherent heterogeneity across included studies, a random-effects model was used for the quantitative analysis of PPD and CAL. Meta-analysis of PPD reduction revealed statistically non-significant weighted mean difference of 0.11 mm (p = 0.08), similarly, there was no statistically significant difference in the mean changes for CAL gain of 0.08 mm (p = 0.39) between APDs and conventional therapy.

In short, it was found that repeated treatment with APDs during SPT offer similar clinical treatment outcomes when compared to the conventional treatment. This signifies that both treatment modalities appear to have similar capability in effectively removing subgingival biofilm and reducing residual PPD, besides achieving comparable CAL gain. This finding is also in agreement with a systematic review reporting effects on clinical parameters with or without repeated intervention of APDs versus conventional methods [24].

#### Patient reported outcomes

Regarding patients’ perception during treatment, statistically significant lower pain score in favour of APDs was observed [15, 18]. Furthermore, APD was the most widely preferred option over conventional therapy for the subsequent follow-up treatment [14]. The discomfort of treatment could be explained from two perspectives: (i) dentine hypersensitivity due to considerable cementum loss following repeated mechanical instrumentation using curettes and/or power-driven scalers [7, 8, 48], and (ii) ulceration to the surrounding soft tissue following use of hand instrument [49]. Hence, one great advantage of APDs is its negligible risk towards irreversible hard and soft tissue damage when used in conjunction with low abrasive powder [23]. This suggests that APDs may be more patient-friendly when compared to other forms of mechanical debridement. This assumption is in line with other systematic reviews on APDs in SPT patients [22–24].

### Key findings in implant maintenance studies

#### Clinical parameters

Inconsistent results were obtained between the two implant studies. Lupi et al. [17] reported a statistically significant higher PPD reduction but non-significant CAL gain in the APD group. With regards to BOP, statistically significant difference between groups at baseline was reported. An issue with randomisation process was highly suspected, accompanied by a high risk of bias. However, if these findings were to be elucidated separately, statistically significant decrease in BOP by 25% and 14% were reported in APD and conventional therapy at 6 months, respectively. It could be concluded that APDs are clinically more effective than the traditional treatment in controlling inflammation. On the other hand, Ziebolz et al. [21] observed no statistically significant changes in PPD and BOP in all preventive approaches, except for the group receiving adjunctive air polishing without chlorhexidine varnish. A significant increase of PPD was reported but this was not considered pathological, given the value lay within a non-diseased range (≤ 5 mm). In addition, there was no concurrent increase in BOP; a finding in line with the case definition and diagnostic consideration for peri-implant health [50].

Thus, these observations indicate that APDs and conventional treatment can be used successfully to prevent peri-implant inflammation in terms of BOP reduction. In addition, although not evaluated in this systematic review, there is also a possibility for combined therapy to prevent peri-implant inflammation. This conclusion is in agreement with the evidence-based recommendations which support mechanical debridement of the implant surface irrespective of type of instrument used for therapy of peri-implant mucositis [51, 52].

### Overall completeness and applicability of the evidence

#### Absence of economic data

In this systematic review, none of the included studies reported on the cost of repeated interventions on both natural dentition and around dental implants. As a result, further economic evaluation could not be conducted, and the cost-effectiveness of those treatment modalities failed to be estimated. Economic evaluation would enable us to determine if the improvement in treatment outcomes based on the latest treatment option is worth the added cost compared to the conventional treatment method.

#### Lack of repeated evaluation of patients’ perception

The evaluation of tolerance to treatment was done only once which was at baseline immediately after the intervention. Hence, effect of repeated treatment on patient’s preference in subsequent follow-up visits remain unclear. Given motivation was reported to be one of the main patient-reported reasons for being non-compliant [53], pleasant experience with no or minimal discomfort is therefore important in order to enhance patient’s motivation and improve their adherence to recall visits.

#### Insufficient information on APDs for implant maintenance

Literature comparing efficacy of repeated intervention using APDs as monotherapy with other conventional preventive approaches in maintaining peri-implant health is scarce. The limited number of studies that were eligible to be included in this review could be explained by the fact that the indications for the usage of APDs in implant dentistry is a relatively recent development. Moreover, patients’ perception cannot be assessed in this group of subjects due to lack of information. Consequently, the conclusion made on the use of APDs during implant maintenance were not based on quantitative data.

### Overall quality, strength and consistency of the evidence

#### Limitations of included studies

The revised Cochrane RoB-2 tool demonstrated that none of the included studies were judged as having a low risk of bias. 4 out of 6 studies (2 SPT and 2 implant studies) were assigned as high risk of bias while the remaining 2 SPT studies had some concerns of bias. Most of the information on concealment of allocation sequence as well as patient- and operator-blinding were not met. Data of dropouts was omitted in two studies [15, 21] and per protocol analysis was used instead to estimate the intervention effect. Therefore, the overall quality of the evidence in the present review must be considered.

The consistency of evidence is another issue. In three studies [13, 15, 18], inclusion of smokers may have influenced the clinical parameters and caused poorer response as smoking can affect the outcomes of non-surgical periodontal treatment [54–57]. Poor reporting with respect to the number of pack years along with the amount of cigarette consumption could further complicate the interpretation of the study results [58, 59]. Besides that, the tested sites in the SPT studies consist of a mixture of single-rooted and multi-rooted teeth, with furcation involvement not specified as an exclusion criterion in some studies. It is well established that presence of residual pockets at multi-rooted teeth may influence the treatment outcomes [54, 57, 60]. With regards to study designs, the carry-across effects in RCTs adopting the split-mouth approach cannot be ruled out and may induce bias in treatment efficacy [61]. Nevertheless, special nozzles were used in the test group of most studies to allow access to the subgingival area, the depth of debridement was equalised with the control group and the spill of air polishing powders to the control site was also minimised. Thus, it can be assumed that the carry-across effect is minimised, probably to a negligible level.

#### Strengths of the review

Despite the aforementioned shortcomings, this systematic review is the first in assessing clinical outcomes and patients’ perception following repeated interventions in both SPT and implant maintenance. Treatment of APDs or conventional therapy were carried out at least twice throughout the entire observation period of at least six months duration. Previous systematic reviews had largely ignored the time factor and incorporated studies with follow-up duration as short as 7 days [24] or 3 weeks [25]. Most included studies also have similar 3-month interval of retreatment. In addition, this review highlighted the lack of economic evaluation of treatment provided in SPT and implant maintenance patients. Ultimately, having a cost estimation of a treatment modality is not a decisive factor in opting for the latest treatment modality but to evaluate the cost-effectiveness of this treatment over the course of the visits. This will allow optimal allocation of funding in the public sector providing specialist periodontal treatment and simultaneously determine whether the clinical benefits of that treatment are worth the added cost.

### Potential biases in the review process

In order to minimise potential bias throughout the review process, study selection, data extraction and risk of bias assessment were carried out by two reviewers independently. The search was also designed following stringent criteria and highly sensitive electronic search of multiple databases, as well as grey literature supplemented with hand searching was performed However, only full-text articles published in the English language were retrieved for assessment of eligibility. It has been shown that the precision of pooled estimates improved with the inclusion of results from non-English language studies [62]. Although English language is generally perceived to be the universal language of science, excluding languages other than English may introduce a language bias and may lead to inaccurate conclusions.

### Implications for practice and policy

Within the limitations of the research, the data shows that repeated interventions using APDs in SPT patients resulted in similar clinical outcome for PPD reduction but was associated with lower pain score and higher patient acceptance. In terms of implant maintenance, APDs resulted in promising clinical outcomes for PPD and BOP reduction. Hence, APDs may be used as an alternative to conventional mechanical debridement in periodic maintenance of periodontal and peri-implant mucosal health in SPT and implant patients.

### Implications for future research

There are several suggestions for future directions on research of APDs in SPT as well as in implant maintenance patients in order to improve the overall quality and consistency of evidence:

- Population and study design
  - To exclude current smokers from the studies.
  - To investigate effects of repeated intervention with longer follow-up duration of at least 12-month.
- Objectives
  - To standardise assessment of clinical parameters.
  - To include microbiological assessment to support clinical outcomes.
  - To conduct economic evaluation on types of treatment modality used.
  - To investigate the effects on multi-rooted teeth with or without furcation involvement.
  - To assess patients’ comfort by using VAS scale as a tool at multiple intervals.
  - To assess tooth/implant loss (survival rate) as one of the tangible outcomes.

## Conclusions

Within the limitations of this systematic review and meta-analysis, the following conclusions can be drawn:

1. Repeated subgingival debridement using APDs resulted in similar clinical outcomes in terms of PPD reduction when compared to hand scaling and/or power-driven instruments in SPT patients.
2. Current evidence shows that subgingival debridement using APDs has better patients’ reported outcomes compared to hand scaling and/or power-driven instruments in SPT patients.
3. Repeated subgingival debridement using APDs might have potential in improving clinical outcomes compared to hand scaling and/or power-driven instruments in implant maintenance patients.

## Supplementary Information

**Additional file 1**. PRISMA Checklist.

**Additional file 2**. Search Strategy.

## Data Availability

The datasets used and analysed in this review are available from the corresponding author on reasonable request.

## Abbreviations

SPT: Supportive periodontal therapy
APDs: Air polishing devices
RoB 2: Revised cochrane risk-of-bias tool
PPD: Probing pocket depth
WMD: Weighted mean difference
BOP: Bleeding on probing
CAL: Clinical attachment level
VAS: Visual analogue scale
PICO: Population, intervention, comparison, outcomes
PROMs: Patient reported outcome measures
QoL: Quality of life
RCTs: Randomised controlled clinical trials
SD: Standard deviation
SEM: Standard error of the mean
