# Supplementary material for: Efficacy of air polishing in comparison with hand instruments and/or power-driven instruments in supportive periodontal therapy and implant maintenance: a systematic review and meta-analysis
Source: BMC Oral Health. 2022 Mar 23;22:85. doi: 10.1186/s12903-022-02120-6 (PMC8944123; doi:10.1186/s12903-022-02120-6)
Supplement: Supplementary file 2 — Additional file 2. Search Strategy. [file 12903_2022_2120_MOESM2_ESM.docx]

**SUPPLEMENTARY DOCUMENT**

# ADDITIONAL FILE 2: SEARCH STRATEGY

| **Database** | **Search Terms** | **Search Results** |
| --- | --- | --- |
| Cochrane Library – Cochrane Central Register of Controlled Trials | 1. (periodontitis OR “periodontal diseases” OR “chronic periodontitis” OR “aggressive periodontitis” OR “periodontal pocket” OR peri-implant* OR “peri-implant *” OR implant*):ti,ab,kw   AND   1. (“air abrasion, dental” OR “air abrasive*” OR “air polish*” OR “air polishing device*” OR airflow OR “dental polishing” OR “dental prophylaxis” OR “glycine powder” OR “erythritol powder” OR “sodium bicarbonate powder”):ti,ab,kw   AND   1. (“periodontal debridement” OR “dental scaling” OR “root planing” OR “periodontal treatment” OR “periodontal therapy” OR “root surface debridement” OR “conventional debridement” OR “conventional treatment” OR “conventional therapy” OR “non-surgical treatment” OR “non-surgical therapy” OR “nonsurgical treatment” OR “nonsurgical therapy” OR “universal curette*” OR curette* OR “Gracey curette” OR “manual scal*” OR “hand scal*” OR “hand instrument*” OR “ultrasonic scal*” OR “ultrasonic instrument*”):ti,ab,kw | 96 |
| PubMed | 1. periodontitis[Title/Abstract] OR periodontal diseases[Title/Abstract] OR chronic periodontitis[Title/Abstract] OR aggressive periodontitis[Title/Abstract] OR periodontal pocket[Title/Abstract] OR peri-implant*[Title/Abstract] OR peri-implant *[Title/Abstract] OR implant*[Title/Abstract]   AND   1. air abrasion, dental[Title/Abstract] OR air abrasive*[Title/Abstract] OR air polish*[Title/Abstract] OR air polishing device*[Title/Abstract] OR airflow[Title/Abstract] OR dental polishing[Title/Abstract] OR dental prophylaxis[Title/Abstract] OR glycine powder[Title/Abstract] OR erythritol powder[Title/Abstract] OR sodium bicarbonate powder[Title/Abstract]   AND   1. periodontal debridement[Title/Abstract] OR dental scaling[Title/Abstract] OR root planing[Title/Abstract] OR periodontal treatment[Title/Abstract] OR periodontal therapy[Title/Abstract] OR root surface debridement[Title/Abstract] OR conventional debridement[Title/Abstract] OR conventional treatment[Title/Abstract] OR conventional therapy[Title/Abstract] OR non-surgical treatment[Title/Abstract] OR non-surgical therapy[Title/Abstract] OR nonsurgical treatment[Title/Abstract] OR nonsurgical therapy[Title/Abstract] OR universal curette*[Title/Abstract] OR curette*[Title/Abstract] OR Gracey curette[Title/Abstract] OR manual scal*[Title/Abstract] OR hand scal*[Title/Abstract] OR hand instrument*[Title/Abstract] OR ultrasonic scal*[Title/Abstract] OR ultrasonic instrument*[Title/Abstract] | 74 |
| EMBASE | 1. (periodontitis OR periodontal diseases OR chronic periodontitis OR aggressive periodontitis OR periodontal pocket OR peri-implant* OR peri-implant * OR implant*). [All field]   AND   1. (air abrasion, dental OR air abrasive* OR air polish* OR air polishing device* OR airflow OR dental polishing OR dental prophylaxis OR glycine powder OR erythritol powder OR sodium bicarbonate powder). [All field]   AND   1. (periodontal debridement OR dental scaling OR root planing OR periodontal treatment OR periodontal therapy OR root surface debridement OR conventional debridement OR conventional treatment OR conventional therapy OR non-surgical treatment OR non-surgical therapy OR nonsurgical treatment OR nonsurgical therapy OR universal curette* OR curette* OR Gracey curette OR manual scal* OR hand scal* OR hand instrument* OR ultrasonic scal* OR ultrasonic instrument*). [All field] | 136 |
| Web of Science (WoS) | 1. TOPIC: periodontitis OR periodontal diseases OR chronic periodontitis OR aggressive periodontitis OR periodontal pocket OR peri-implant* OR peri-implant * OR implant*   AND   1. TOPIC: air abrasion, dental OR air abrasive* OR air polish* OR air polishing device* OR airflow OR dental polishing OR dental prophylaxis OR glycine powder OR erythritol powder OR sodium bicarbonate powder   AND   1. TOPIC: periodontal debridement OR dental scaling OR root planing OR periodontal treatment OR periodontal therapy OR root surface debridement OR conventional debridement OR conventional treatment OR conventional therapy OR non-surgical treatment OR non-surgical therapy OR nonsurgical treatment OR nonsurgical therapy OR universal curette* OR curette* OR Gracey curette OR manual scal* OR hand scal* OR hand instrument* OR ultrasonic scal* OR ultrasonic instrument* | 366 |
| Dentistry & Oral Sciences Source | 1. AB (periodontitis OR periodontal diseases OR chronic periodontitis OR aggressive periodontitis OR periodontal pocket OR peri-implant* OR peri-implant * OR implant*)   AND   1. AB (air abrasion, dental OR air abrasive* OR air polish* OR air polishing device* OR airflow OR dental polishing OR dental prophylaxis OR glycine powder OR erythritol powder OR sodium bicarbonate powder)   AND   1. AB (periodontal debridement OR dental scaling OR root planing OR periodontal treatment OR periodontal therapy OR root surface debridement OR conventional debridement OR conventional treatment OR conventional therapy OR non-surgical treatment OR non-surgical therapy OR nonsurgical treatment OR nonsurgical therapy OR universal curette* OR curette* OR Gracey curette OR manual scal* OR hand scal* OR hand instrument* OR ultrasonic scal* OR ultrasonic instrument*) | 151 |
